# Development and characterization of a novel mAb against bilitranslocase - a new biomarker of renal carcinoma

**DOI:** 10.2478/raon-2013-0026

**Published:** 2013-05-21

**Authors:** Sendi Montanic, Michela Terdoslavich, Uros Rajcevic, Luigina De Leo, Serena Bonin, Vladka Curin Serbec, Sabina Passamonti

**Affiliations:** 1 Blood Transfusion Centre of Slovenia, Ljubljana, Slovenia; 2 Rottapharm Biotech S.r.l., Research Centre AREA Science Park, Trieste, Italy; 3 Institute of Child Health IRCCS “Burlo Garofolo” Trieste, Trieste, Italy; 4 Department of Medical Sciences, University of Trieste, Cattinara Hospital, Trieste, Italy; 5 Department of Life Sciences, University of Trieste, Trieste, Italy

**Keywords:** bilitranslocase, monoclonal antibody, peptide antigen, kidney, renal cell carcinoma, tumor marker

## Abstract

**Background:**

Bilitranslocase (TC 2.A.65.1.1) is a bilirubin-specific membrane transporter, found on absorptive (stomach and intestine) and excretory (kidney and liver) epithelia and in vascular endothelium. Polyclonal antibodies have been raised in rabbits in the past, using a synthetic peptide corresponding to AA65-77 of rat liver bilitranslocase, as an antigen. Affinity-purified antibodies from immune sera have been found to inhibit various membrane transport functions, including the bilirubin uptake into human hepatocytes and the uptake of some flavonoids into human vascular endothelial cells. It was described by means of immunohistochemistry using polyclonal antibodies that bilitranslocase expression is severely down-regulated in clear cell renal carcinoma. The aim of our work was development and characterization of high-affinity, specific mAbs against bilitranslocase, which can be used as a potential diagnostic tool in renal cell carcinoma as well as in a wide variety of biological assays on different human tissues.

**Materials and methods:**

Mice were immunized with a multi-antigen peptide corresponding to segment 65–75 of predicted primary structure of the bilitranslocase protein. By a sequence of cloning, immune- and functional tests, we aimed at obtaining a specific monoclonal antibody which recognizes a 37 kDa membrane protein, and influences the transport activity of bilitranslocase.

**Results:**

On the basis of previous results, specific IgM monoclonal antibodies were produced in BALB/c mice, in order to further improve and extend the immunological approach to the study of bilitranslocase in renal cancer cells as well as to develop its potential diagnostics use.

**Conclusions:**

In this article we show an immunological approach, based on newly developed monoclonal antibodies, to a detailed biochemical and functional characterization of a protein whose gene and protein structure is still unknown. We were able to demonstrate our novel mAb as a tumor marker candidate of renal cell carcinoma, which may prove useful in the diagnostic procedures.

## Introduction

Bilitranslocase (BTL, TC 2.A.65.1.1) is a plasma membrane transporter originally identified in rat liver.^1^ It transports various polyaromatic compounds, whether endogenous, such as bilirubin, or of plant origin, such as dietary flavonoids. It is expressed in various rat and human tissues, including the gastrointestinal epithelium, the vascular endothelium^2^–^4^ and the kidney.^5^–^7^

Given the unusual feature of BTL coding sequence, which corresponds to the anti-sense strand of a segment of the gene encoding for the plasma protein ceruloplasmin^1^, the only way to assess the expression of this protein in cells and tissues is by immunological approach, using specific anti-peptide antibodies.

A previous investigation using anti-peptide polyclonal antibodies has shown that bilitranslocase expression is severely down-regulated in clear cell renal carcinoma.^8^

The most used anti-peptide, polyclonal antibody, targeting an extracellular domain of mammalian bilitranslocase was produced by immunizing rabbits with a multi-antigen peptide, corresponding to segment 65–75 of the primary structure of rat liver bilitranslocase.

The affinity-purified antibody showed a wide biological activity, recognizing both the denatured protein in immunoblots and inhibiting its function in intact cells and purified plasma membrane fractions.^9^

To further improve and extend the immunological approach to the study of bilitranslocase expression in pathological tissue in kidney cancers, we aimed to produce a monoclonal antibody raised in BALB/c mice that, while keeping the above biological features, would also meet the requirements of indefinite reproducibility, stability and long shelf-life necessary for a reliable diagnostic tool.

Renal cell carcinoma (RCC), the most common neoplasm of the adult kidney accounts for 2–3% of all malignant diseases in adults. It is the seventh most common cancer in men and the ninth most common in women. Global incidence and mortality rates of RCC are rising^10^ with its incidence worldwide of about 209 000 new cases per year and 102 000 deaths per year.^11^ The majority of kidney tumors are of the clear cell (ccRCC) subtype.^12^ Although imaging techniques for abdominal screening have led to the increased incidental detection of renal tumors^13^, unfortunately 25–30% patients still have metastases at presentation. The prognosis of RCC is quite variable. The greatest risk of recurrence following nephrectomy is within the first 3–5 years.^14^ Metastatic renal cell carcinoma (RCC) is one of the most treatment-resistant malignancies and patients have a dismal prognosis with a <10% five-year survival rate. The identification of markers that can predict the potential of metastases will have a great impact in improving the patient’s outcome.^15^–^17^ Currently, the patient’s prognosis is assessed by histological parameters and a multivariate analysis developed at Memorial Sloan Kettering^18^, but neither is sufficiently accurate. A more accurate assessment of the prognosis is urgently needed to better guide the patient’s management. As our preliminary data on bilitranslocase expression in renal cancer compared to corresponding normal tissue showed remarkable differences, we aimed at producing a monoclonal antibody that would meet the requirement of serving as a tool for the assessment of bili-translocase as a novel biomarker of human kidney cancers.

## Materials and methods

### Experimental design

Mice were immunized with a multi-antigen peptide corresponding to segment 65–75 of predicted primary structure of the bilitranslocase protein. By a sequence of cloning, immune- and functional tests, we aimed at obtaining a specific IgM monoclonal antibody, which recognizes a 37 kDa membrane protein, and influences the transport activity of BTL, by inhibiting the transfer of BSP in plasma membrane vesicles. Thus, we aimed at producing a tool suitable to give an insight into both the expression and the functioning of BTL.

### Peptide and peptide conjugate

A multi-antigen peptide corresponding to segment 65–75 of the predicted amino acid sequence of bili-translocase (AA 65–75; EDSQGQHLSSF) was conjugated to keyhole limpet hemocyanin (KLH) by Inject mcKLH Immunogen EDC Conjugation Kit (Pierce).

### Immunization of Balb/c mice and generation of mAbs

Animals were fed with standard laboratory chow (Harlan Teklad) and tap water ad libitum; they were housed in temperature controlled rooms and humidity at the Animal House of the Blood Transfusion Centre of Slovenia (BTCS), Ljubljana, Slovenia. Three female BALB/c mice were immunized subcutaneously with 15 μg of multi-antigen peptide in complete Freund’s adjuvant followed by three intraperitoneal inoculations of 15 μg of the same antigen in incomplete Freund’s adjuvant after 2, 4 and 9 weeks (authorized by Veterinary Administration of the Republic of Slovenia, No.: 3440-63/2006, of 31.7.2006). After 4^th^ immunization, polyclonal sera were collected from the tail vein and antibody titres were estimated with indirect ELISA (as described below). Two mice with the highest antibody titre were boosted with peptide in physiological saline and sacrificed. Fusion of spleen lymphocytes with mouse myeloma NS1 cells was performed by hybridoma technology, according to our protocols. After HAT (hypoxanthine-aminopterine-thymidine) selection, hybridomas producing antibodies of desired specificity were determined by indirect ELISA and cloned by limiting dilution.

### Screening of the sera and hybridoma supernatants by enzyme-linked immunosorbent assay (ELISA)

The presence of specific antibodies in antisera and hybridoma supernatants was checked by indirect ELISA. Microtiter plates (Corning) were coated with 50 μl of peptide (3 μg/ml) in 50 mM carbonate/bicarbonate buffer (pH 9.6), incubated overnight at +4°C, washed with PBS 0.05% Tween20, pH 7.2 (buffer B) and then blocked with 1% BSA (bovine serum albumin) in the same buffer (buffer C). 50 μl of hybridoma supernatant or corresponding serum dilution was added to the wells and incubated for 90 min at +37°C. Negative isotypic controls were carried out by using equivalent amounts of hybridoma supernatants producing mAbs against blood group antigen B. Plates were washed with buffer B and 50 μl of HRP conjugated anti-mouse immunoglobulin (Jackson ImmunoResearch), diluted 1:3000 in buffer C, was added to each well and incubated for 90 min at +37°C. After a final wash in buffer B, 50 μl ABTS substrate (Sigma) was added to each well. The absorbance was measured at 405 nm after 30 min of incubation at +37°C. Titres were evaluated by the end-point dilution approach (the highest dilution of the sera that was still two times higher as the background).

### Purification of monoclonal antibodies

Monoclonal antibodies were purified from the cell culture supernatants by liquid chromatography system FPLC on HiTrap Protein G HP column (GE Healthcare) by affinity chromatography, using 0.1 M glycine, pH 2.7, for elution or HiTrap IgM Purification HP column (GE Healthcare) by thiophilic adsorption on 2-mercaptopyridine, coupled to Sepharose at 1M ammonium sulphate.

### Rat liver subfractions preparation: plasma membrane vesicles, microsome and cytosolic fraction

Subfractions were prepared from Wistar albino female rat liver (250 g). Animals were fed with standard laboratory chow (Harlan Teklad) and tap water *ad libitum*; they were housed in temperature controlled rooms and humidity at the Animal House of the University of Trieste according to the provision of the European Community Council Directive (n.86/609/CEE) and to the provision of Italy (D.L.vo 119/92). For the preparation of plasma membrane vesicles, microsomes and cytosolic fractions, 6 rats were used as described below.

#### Plasma membrane vesicles preparation

Plasma membrane vesicles were prepared from 3 rat livers as described.^7^ The final pellet was suspended in 10 mM Hepes, pH 7.4 /0.25M sucrose, and stored in liquid nitrogen. Vesicles, thawed at +37°C, were used for the bilitranslocase activity assay. When denaturized in SDS-PAGE sample buffer, they were subjected to the western blot analysis.

#### Rat Liver Microsomes and cytosolic subfraction

To get sufficient tissue preparation to perform all assays, three livers (7 g each) were rapidly excised, minced with scissors and washed out the excess of blood with chilled Homogenization Buffer (1 mM EDTA in PBS, pH 7.4). Liver pieces were further suspended in 4 volumes, equivalent to sample weight, of Homogenization Buffer with Protease Inhibitor Cocktail (PIC; Sigma) and homogenized by 30 ml Glass/Teflon grinder, submerged in a small bucket of ice during homogenization. The homogenate was further centrifuged for 15 min at 15.000 g at +4°C. Mitochondrial pellet was discarded and the supernatant was centrifuged at 100.000 g for 60 min at +4°C. The upper lipid layer was removed, the cytosolic supernatant collected (SN2), whereas the microsome pellet was resuspended in Homogenisation Buffer with PIC (Sigma). Both samples (SN2 and microsomes) were aliquoted and stored at −80°C. Protein concentration was measured according to the Bradford method.

### Western blot

Rat plasma membrane microsome and cytosolic preparations were denaturated with SDS-PAGE sample buffer at +100°C for 5 min. 50 μg of sample per lane was loaded on SDS-tris-glycine polyacrilamide gels (9%) and separated by PAGE. The separated proteins were transferred to 0.2 μm nitrocellulose membranes (Whatman) by a standard procedure. The membranes were blocked in 5% non-fat milk and incubated for 90 min at room temperature (RT) with tested hybridoma supernatants, diluted 1:5 in 1% milk/TBS-T, or purified monoclonal antibodies (mAb) at a final concentration of 10 μg/ml. As negative controls were used equivalent amounts of purified monoclonal antibody of the same isotype against blood group antigen B. Membranes were washed with TBS-T and incubated for 90 min at RT with HRP labeled anti-mouse secondary antibody (Jackson ImmunoResearch) 1:5000 in 1% milk/TBS-T. Chemiluminescence detection was performed using ECL reagent (Amersham Biosciences).

### Cell culture

Human hepatoblastoma HepG2 cell line was obtained from American Type Culture Collection (Rockville, MD, USA) and maintained in Eagle’s Minimum Essential Medium (Sigma), supplemented with 2 mM glutamine, 1mM Sodium pyruvate, 10% (v/v) fetal bovine serum, 100 μg/ml streptomycin and 100 U/ml penicillin (Sigma). Cells were grown in a humidified incubator at 5% CO_2_ and +37°C. Cells for the flow-cytometry analysis were detached from the flasks after 6 days of culture with 20 mM EDTA and used for the experiment. Alternatively, cells were seeded on cover glasses and after 4 days of culture, they were fixed with 2% paraformaldehyde, being ready for the immunocytochemistry assay.

### Flow cytometry

For each sample, 5×10^5^ HepG2 cells were first washed with staining solution (1% FCS, 0.02% Na-azide in PBS), stained with primary mAb for 30 min at +4°C, then washed again with staining solution and incubated with secondary antibody (Sigma), diluted 1:50 for 30 min at +4°C. After staining, cells were washed three times and resuspended in 300 μl of staining solution. Detection was immediately performed on a flow cytometer (Becton Dickinson). Polyclonal rabbit antibodies were used as a positive control. For the intracellular analysis, the cells were first fixed with 4% para-formaldehyde and permeabilised with permeabilisation buffer (0.5% saponine, 5% BSA, 0.02% Sodium Azide in PBS).

### Immunocytochemistry

HepG2 cells, fixed on the cover glasses, were permeabilised with 0.2% Triton (Sigma) and blocked with blocking solution (5% normal goat serum (Sigma) and 4% BSA (Sigma) in PBS (Sigma) for 1 hour at +37°C. The staining with primary antibodies was performed at a final concentration of 50 μg/ ml overnight at +4°C. Negative controls were obtained by using purified monoclonal antibody of the same isotype against blood group antigen B. Next day, the cells were incubated with secondary antibodies (Sigma), diluted 1:100 in blocking solution for 1 h at +37°C. Cell nuclei were stained by incubating with 1 μg/ml DAPI (Sigma; 4–6-diamidino-2 phenylindole) in 1% Tween-PBS for 5 min at RT. Between all incubation steps, cells were always washed with PBS. Finally, cells were dehydrated with 50%, 85% and 96% ethanol and mounted with 1.4-diazabicyclo (2.2.2.) octane (DABCO; Sigma) on microscope slides.

### Immunohistochemistry

Immunohistochemistry (IHC) was performed on 3 μm tissue sections of renal cancer carcinoma tissue section and a normal human kidney tissue section according to standard procedures. The immunostaining was performed with the Vectastain Universal Elite ABC kit (Vector Laboratories). Briefly, after dewaxing and rehydration, slides were treated with H_2_O_2_ and blocked by Vectastain Elite ABC kit (Vector Laboratories) for 20 min. Incubation with the primary antibody was performed at RT for 1 hour (2 μg/ml in PBS containing 0.1% BSA). After washings in PBST, the slides were incubated for 30 min with the biotinylated secondary antibody and submitted to ABC kit for the staining development. As described by the manufacturer, IHC was performed with DAB as chromogen and hematoxylin as counterstain. To investigate the specificity of the primary mAb in IHC detection, the primary antibody solution (1 ml) was added on HepG2 monolayers grown on 75 cm^2^ flasks for 30 min at RT. Then, the same antibody solution was transferred to another flask of HepG2 cells monolayer and again incubated for 30 min. This procedure was repeated once more. This pre-adsorbed antibody solution was tested on tissue sections, where staining was negligible.

### Bilitranslocase transport activity assay

The transport activity of BTL was characterized in rat liver plasma membrane vesicles by real-time dual wavelength spectrophotometric recording of BSP (sulfobromophtalein) disappearance from the medium into the vesicles at 580 nm–514 nm.^19^

### Electrogenic BSP uptake inhibition by antibodies

The kinetics of bilitranslocase transport activity inhibition by antibodies was examined as described^19^ by pre-incubation of rat liver plasma membrane vesicles at +37°C with antibody. Controls were carried out by using equivalent amounts of purified monoclonal antibody against blood group antigen B. Aliquots of the pre-incubation mixture were withdrawn during a 15 min span and added to the transport medium for the assay of bilitranslocase transport activity.

### Data analyses

As previously described^19^, the extent of inactivation of each time sample was expressed as T_I_/T_0_ (relative transport activity), where T_0_ and T_I_ are transport activities, measured in the absence (or at time zero) or in the presence of the antibody, respectively. The following equation was fitted to data: *y = y*_0_+*ae*^−kt^, where *y* = T_I_/T_0_, *y*_0_ = residual T_I_/T_0_ at steady state, *a* = 1−*y*_0_, *e* = 2.7183, *t* = time and *k* = inactivation rate constant (min^−1^). Thus, the inactivation rate constants parameters in the absence (*k*_0_) and in the presence of increasing bilirubin concentrations (kS1, kS2, … kSn) were obtained. The inhibition rate constants can be related to bilirubin concentration by the Scrutton & Utter equation^20^

kS/k0=k2/k1+Kd(1-(kA/k0))/(S)

where kS and k0 are the inactivation rate constants either in the presence or in the absence of various concentrations of a bilirubin, k2 and k1 are the rate constants of the inhibition of the bilitranslocase-bilirubin complex and of free bilitranslocase, respectively. Kd is the dissociation constant of the apparent bilitranslocase-bilirubin complex.

Data for the characterization of the kinetics of electrogenic BSP uptake fitted the Michaelis-Menten equation and the apparent Km and Vmax values were derived with their standard errors. Data were analyzed by means of SigmaPlot 2001 (SPSS Science Software Gmbh).

### Statistics

**FIGURE 1:**
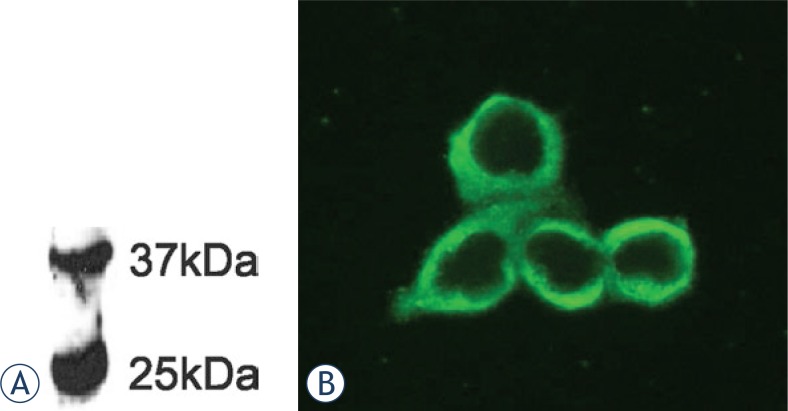
Application of purified anti-BTL mAb in WB and ICC assays. (A) WB of rat liver denatured microsome (M), performed with purified mAb 6E4/1F2/1E2 (10μg/ml). (B) ICC staining of HepG2 cells with mAb 6E4/1F2/1E2 (10μg/ml).

Immunoblotting experiments were performed 3 times, obtaining consistent results. Figure 1A displays the most successful one (less background, optimal protein load, optimal primary and secondary antibody dilutions and time of exposure).

The transport activity assay was performed on a single vesicle preparation. Tests were done in quintuplicate in preliminary experiments (not shown), testing the dependence of BSP uptake rate on either protein or valinomycin. The coefficients of variations were always below 2%. All measurements, displayed in Figure 3A, B, C, are single. To assess the reproducibility of the parameters of the best fitting curves, experiments were performed at least three times.

## Results

According to our previous results, the immunogenic epitope of interest is segment 65–75; EDSQGQHLSSF of the predicted primary structure of bilitranslocase. Polyclonal antibodies against such epitope already showed multiple biological activities, *i.e.* to react with 1) the protein, expressed in *E. coli*, 2) a single protein in rat liver microsomes, separated by SDS-PAGE electrophoresis and 3) the purified rat liver bilitranslocase.^13^ The same antibodies also inhibited a) the BSP electrogenic uptake in rat liver plasma membrane vesicles, b) the BSP electrogenic uptake in microsomes obtained from HepG2 cells and c) the uptake of bilirubin and BSP into HepG2 cells.^9^,^21^ These properties justified the development of a mAb raised against the same peptide in order to produce a reproducible and unlimited tool.

### Testing of the immune response against peptide, preliminary screening and cloning

The polyclonal immune response to peptide EDSQGQHLSSF was tested by ELISA. Immunization resulted in a strong immune response in all injected BALB/c mice. The mouse with the highest anti-peptide titre was chosen for the cell fusion. Two separate cell fusions of mouse splenocytes with NS1 mouse myeloma cells were performed.

Over 3000 hybridomas were obtained after the cell fusion and, after screening by ELISA, 67 clones produced antibodies, which reacted with the chosen peptide, but not with KLH. The second step of the selection was done by ELISA, using rat liver microsomes as antigen for coating the plate. The results restricted the number of positive clones to 22. The antibodies, produced by selected hybridoma cell lines, were also analyzed in western blot (WB), checking their binding capacity to rat liver microsome (M) or cytosolic fraction (SN2) denatured preparations (data not shown).

Selected hybridomas, which also satisfied cell culture freezing, growing features and production of stable mAbs, were chosen for cloning and additional screening. The first step of the screening confirmed the results, obtained before cloning of hybridomas by repeating ELISA and WB analysis. Fluorescence-activated cell sorting (FACS) and immunocytochemistry (ICC) were carried out as *in vitro* assays for the further antibody characterization. Selected antibodies recognized a protein with MW 37 kDa, both in microsomes rat liver and cytosolic preparations (data not shown). Clone 6E4/1F2 was the best candidate in our early selection criteria.

### Final screening, antibody characterization and applications

Cell line 6E4/1F2 was further cloned and mAbs were purified. All ELISA/WB/FACS/ICC tests were repeated as described above and lead to the finally selected mAb, named 6E4/1F2/1E2, produced by stable hybridoma cell line.

WB analysis, performed with purified mAb, as shown in Figure 1A, confirmed the binding to a protein with MW around 37 kDa. The purified mAb 6E4/1F2/1E2 was tested also in immunocytochemistry (Figure 1B) and as it is shown in this figure, the antibody formed immune complexes on the surface of fixed HepG2 cells.

Our findings show that the selected mAb displays the required features of selectivity and specificity of binding to BTL.

**FIGURE 2:**
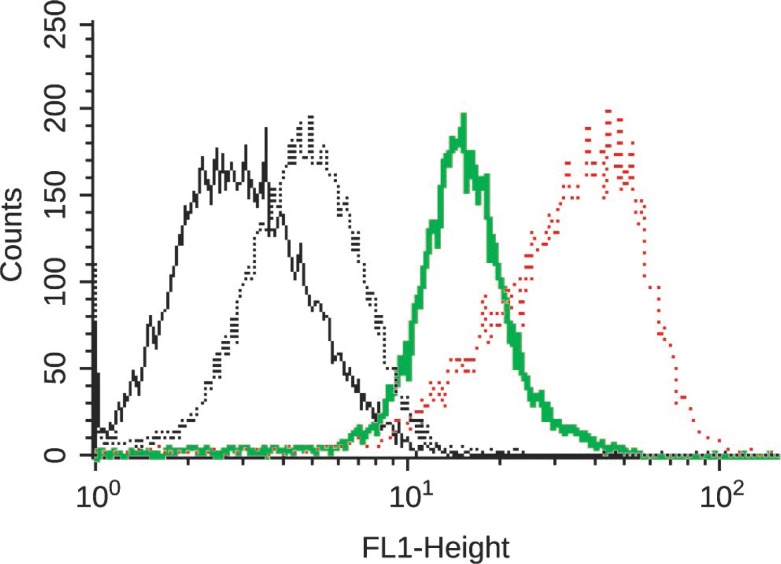
Application of purified anti-BTL mAb in FACS. Reactivity of mAb 6E4/1F2/1E2 with native BTL, expressed on HepG2 cells, determined by FACS as follows: cells only (black line), cells with secondary antibody (black dotted line), intracellular staining (green line), surface staining (red dotted line).

FACS analyses were carried out including both, intracellular and surface protocols. Fixed HepG2 cells were used only for intracellular staining, whereas surface staining was applied on non-fixed cells in order to limit any possible antigen damage due to the fixation procedure. This strategy was applied in order to confirm the apparent BTL localization, derived from ICC results. Figure 2 shows, as expected, prevalent extracellular staining.

**FIGURE 3:**
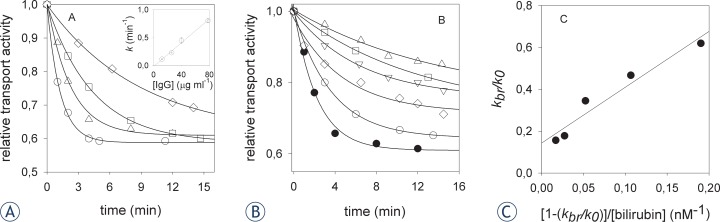
Application of purified anti-BTL mAb in protein activity inhibition. (A) Time-dependent inhibition of electrogenic BSP transport activity in rat liver plasma membrane vesicles by mAb 6E4/1F2/1E2. Vesicles (3.6 mg protein/ml) were pre-incubated with mAb 6E4/1F2/1E2 (μg/ml) as follows: 12 (♦), 26 (□), 40 (▵), 78 (○) at 37°C. Aliquots (3.5 μl) were assayed for electrogenic BSP transport (24 μM BSP). The calculated second order rate k was 0.01±0.00 min-1 ml μg-1; r2=0.99. (B) Time-dependent inhibition of electrogenic BSP transport activity in rat liver plasma membrane vesicles by mAb 6E4/1F2/1E2 tested in the presence of bilirubin. Vesicles were pre-incubated with mAb 6E4/1F2/1E2 (40μg/ml) in absence or in the presence of bilirubin, as follows (nM): 2 (○), 5 (♦), 12.5 (▿), 30 (□), 50 (▵) at 37°C. Aliquots (3.5 μl) were assayed for electrogenic BSP transport (24 μM BSP). (C) Scrutton & Utter plot. The parameters of the regression curve are: y_0_ = 0.143±0.04; slope = K_d_ = 2.67±0.38 nM; r^2^=0.94. Best-fitting curves to the data were obtained as described in Materials and methods.

The antibody was also tested for its inhibition of electrogenic bromosulphalein (BSP) transport in rat liver plasma membrane vesicles, a specific assay of bilitranslocase transport activity. Inhibition was time-dependent (Figure 3A) and linearly dependent on antibody concentration in the range tested (Figure 3A, inset). Two other clones of the cell line 6E4/1F2, *i.e.* 1C8 and 2A8, were also included in testing. Clone 2A8 was inactive. Clone 1C8 displayed a lower inhibition capacity than 1E2, the second order rate constant of inhibition of the latter being 1.4-fold higher than the former.

To check if the apparent transport inhibition observed might be due to an unspecific change of vesicles conductivity, the following experiment was carried out.

Vesicles (2.4 mg/ml in 0.25 M sucrose and 10 mM Hepes pH 7.4) were incubated with the antibodies (12 *μg*/ml) at +37°C for 30 min. Then, they were diluted twice in 0.15 M potassium phosphate buffer pH 8.0 at +20°C and assayed for the electrogenic BSP uptake immediately after dilution and then after 1, 2 and 4 min. In case of disruption of the membrane conductivity, it should be expected that K^+^ would move from the medium into the vesicular compartment, whereas H^+^ would move out from the vesicles (pH 7.4) to the medium (pH 8.0). So, the electrogenic BSP transport activity should be abolished, due to the collapse of the driving forces of BSP movement and accumulation into the vesicles, *i.e*. K^+^ diffusion membrane potential and pH.^11^ It was found that the transport activity was stable for 2 min following the addition of the potassium phosphate buffer pH 8.0 (0.68±0.01 of control) and decreased insignificantly at 4 min (from 0.68±0.01 to 0.66±0.02). This set of results indicates that the antibody changed neither the K^+^ nor the H^+^ conductivity. Otherwise, a drastic and instantaneous effect of the assay should have occurred, *i.e*. a significant decrease of the transport rate.

To further assess if the antibody targeted a bilirubin-binding epitope, exposed on the surface on plasma membrane vesicles, inhibition tests were carried out in the presence of increasing concentrations of bilirubin, in the range 0 nM–50 nM. As previously observed, bilirubin caused the inhibition reaction to proceed at a slower rate (Figure 3B,C). The rearrangement of the data by the Scrutton & Utter equation enabled the derivation of the dissociation constant of the transporter-bilirubin complex (K_d_=2.67±0.38 nM), which was similar to the one, previously obtained with polyclonal anti-sequence antibodies. It might be argued that the slower inactivation rate, observed in the presence of bilirubin might be ascribed to an interaction of the latter with the hypervariable region of the immunoglobulins, thus preventing antibody complexation with the protein epitope. A control experiment was, therefore, carried out to test this hypothesis. The antibody and bilirubin (50 nM) were pre-incubated at +37°C and then used to test the rate of BSP transport inactivation. No significant change of the rate constant could be observed (data not shown). However, the large decrease in the pseudo-first order rate constant of transport inactivation observed (dropping from 0.4456 min^−1^ in the absence of bilirubin to 0.0695 min^−1^ with 50 nM bilirubin) cannot be accounted for by a sequestration of the antibody by bilirubin, since, under the prevailing experimental conditions, mAb was in 5-fold molar or 10-fold equivalent excess, respectively, in favor of the highest bilirubin concentration tested, *i.e*. 50 nM. Even by assuming that bilirubin complexed with the antibody so reducing its effective concentration by an over-estimated 20%, this should be revealed by a decrease of the pseudo-first order rate constant of transport inactivation from ca. 0.45 min^−1^ to ca. 0.35 min^−1^, as predicted by the value of the second-order rate constant obtained from the experiment shown in Figure 3. However, 50 nM bilirubin caused a 5-fold higher decrease of the first-order rate constant (i.e. 0.0695 min^−1^).

**FIGURE 4:**
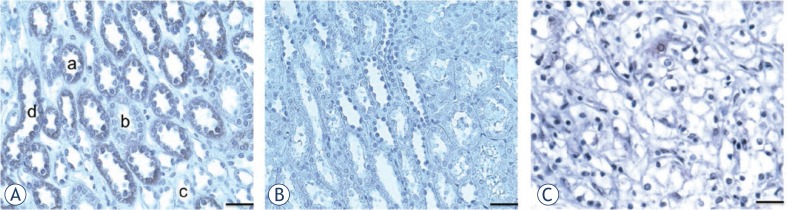
Application of purified anti-BTL mAb in IHC on human RCC and normal kidney tissue sections.

The use of this antibody in immunohistochemistry was tested on human early stage (grade I.) RCC and on normal kidney. Figure 4A shows that the antibody selectively stained distal tubules and the collecting tubules, but none of the segments of the Henle loop. The proximal tubules were also positive (not shown), whereas the vascular structures were negative (not shown). Significantly, staining was nearly abolished (Figure 4B) by pre-adsorption of the antibody solution on a monolayer of HepG2 cells. This outcome was expected, since the immunogenic peptide is a part of an extra-cellular domain of hepatic BTL (Figure 2).^9^ On the opposite, the staining of the grade I RCC tissue section (Figure 4C) was negative.

## Discussion

### Bioactivity of the bilitranslocase mAb

Data obtained by numerous immunological methods show that we selected an IgM mAb, specific for a bilirubin-binding membrane protein. This mAb upgraded properties of our previously tested polyclonal antibodies, with unlimited source and mono-specific binding, binding not only unfolded protein, but also a native one, where it causes a functional loss (Figure 3). Such noteworthy property depends on the fact that antibody binds to an extracellular epitope involved in the transport cycle of the protein. Thus, antibodies targeting that epitope enable to follow not just the expression but also the function of bilitranslocase in viable experimental models, endowed with increasing morphologic complexity, as best seen in vascular endothelial cells.^2^,^4^

Such dual activity of the mAb is particularly advantageous, since bilitranslocase antibodies are currently the only probes to study the occurrence and the function of bilitranslocase. This transporter is, indeed, encoded by an antisense transcript, disabling its detection by current molecular biology tools and approaches. As a consequence, sense-antisense overlaps limit the choice of primers and probes and antisense RNA has usually very low expression levels.^22^ The coding sequence of bilitranslocase (GenBank: Y12178.1) is 94% homologous to a segment of the antisense strand of the ceruloplasmin transcript, including part of its 3’-UTR (NM_012532, segment 2621–3637). The bilitranslocase-ceruloplasmin sense-antisense pair is classified as entry RNO 30106 in the anti-CODE database (http://www.anticode.org) that collects these RNAs. Antisense RNAs are usually non-coding RNAs, though the bilitranslocase transcript has mRNA-like characteristics.^1^,^21^

In cell biology, the correlation between down-regulation of the expression of a protein, by either RNA interference or gene deletion, and decreased cellular function, represents an essential piece of evidence. However, with proteins, translated from antisense transcripts, this cannot be accomplished by the currently validated approaches. Thus, it is clear that antibodies, especially if endowed with the abovementioned dual properties, still represent the reagents of election to track and functionally characterize the so far rare proteins, translated from antisense transcripts.

### Bilitranslocase as a negative tumor marker

Our preliminary findings by immunohistochemical tests showed that bilitranslocase was severely down-regulated in grade I clear cell kidney cancer (cc RCC) compared to normal kidney tissue. The reason for such difference is currently unknown. Further investigations are needed to verify if it is associated to this renal cancer specific histological type and/or to renal cancer aggressiveness. Anyway, bilitranslocase could be inactivated in cancerous tissue for various reasons. This may point at bilitranslocase involvement in tumor suppressor activity and thus its inactivation potentially contributing to tumorigenesis in kidney tissue. Further analyses are needed to confirm this hypothesis.

### Further Applications of the anti-bilitranslocase mAb

#### Modulation of expression

The mAb will be an invaluable tool for the study of the expression of bilitranslocase in various stages of rat embryogenesis and cell differentiation. In the intestinal epithelium, the onset of bilitranslocase expression appears to coincide with the differentiation of stem cells into mature epithelial cells.^1^ This observation would justify the employment of bilitranslocase mAb to mark the stages of normal differentiation as well as of de-differentiation in carcinogenesis.

#### Tissue and cell distribution of bilitranslocase and its assessment as a membrane transporter

Immunological assays allow the identification of the presence of bilitranslocase in tissues and cells, in both animal and plant species, helping to infer aspects of organ physiology depending on the bilitranslocase function. The expression of bili-translocase in both absorptive (gastro-intestinal) and excretory (liver and kidney) epithelia as well as in the vascular endothelium makes it a tremendously interesting drug target. As shown above, bilitranslocase is assessed in plasma membrane vesicles as a BSP electrogenic transporter.^19^ This activity is competitively inhibited by various compounds, suggesting that they cannot only bind into the transport site of the protein, but also be directly transported. The latter demonstration can be obtained in isolated cultured cells, where the uptake of a given molecule is inhibited by cells pre-treatment with bilitranslocase antibodies.^2^,^9^ This antibody-based approach to the study of drug transport can be implemented in normal cells and tissues, without gene or RNA manipulation, which unbalance the transcription level of a protein, or counterbalance on the expression level of functionally subsidiary proteins, or disrupt the intracellular network of protein-protein interactions. For these features, the immunological approach to the study of other drug and nutrient transporters might become standard.

## Conclusions

In conclusion, the described mAb might be used as a research tool to assess bilitranslocase as a marker of transition from normal tissue to its neoplastic transformation in human kidney. In further studies, this antibody could be developed as a diagnostic reagent for kidney cancer. However, this mAb will enable us to screen a variety of tissue samples to prove its use in diagnostics.
